# Draft Genome Sequences of Three *Clostridia* Isolates Involved in Lactate-Based Chain Elongation

**DOI:** 10.1128/MRA.00679-20

**Published:** 2020-08-06

**Authors:** Bin Liu, Denny Popp, Heike Sträuber, Hauke Harms, Sabine Kleinsteuber

**Affiliations:** a Department of Environmental Microbiology, Helmholtz Centre for Environmental Research–UFZ, Leipzig, Germany; Loyola University Chicago

## Abstract

Hitherto, few species have been reported to convert lactate to *n*-caproate. Here, we report the high-quality draft genomes of three *Clostridia* strains isolated on lactate as the sole carbon source. The genomes were assembled using a hybrid short- and long-read sequencing approach. The genes involved in lactate-based chain elongation were identified.

## ANNOUNCEMENT

Recently, we reported reactor microbiota that produce *n*-caproate from corn silage by anaerobic fermentation (1). To enrich the lactate-consuming bacteria involved in this process, anaerobic batch cultures in liquid mineral medium with lactate as the sole carbon source were inoculated with sieved reactor broth (mesh size, 2 mm; inoculation ratio, 1:10) from a lab-scale continuous stirred tank reactor (fermenting corn silage at 38°C; pH, 5.5; hydraulic retention time, 4 days) and incubated at 37°C. Pure strains from single colonies were isolated on agar medium DSM 104c with 5 g/liter lactate; their fermentation products were analyzed in liquid culture. Strains were identified by PCR and Sanger sequencing using 16S rRNA-specific primers (2). Three caproate-producing isolates designated BL-3, BL-4, and BL-6 represented new species based on their 16S rRNA gene sequences (3) and were selected for whole-genome sequencing (WGS).

Genomic DNA was extracted from the cell pellets using a NucleoSpin microbial DNA kit (Macherey-Nagel, Germany). WGS was performed with both long and short reads to obtain accurate sequences and complete scaffolds. Short-read sequencing using the Illumina NextSeq 500 system (NEBNext Ultra II FS DNA library prep kit; 2 × 150 bp) was performed by StarSEQ GmbH (Mainz, Germany). FASTQ data generation, demultiplexing, and adapter trimming of the raw sequencing reads were automatically performed by the Illumina software. The sequence quality was analyzed using FastQC v0.11.9 (4). For long-read sequencing, the library was prepared using the ligation sequencing kit (1D SQK-LSK109) and the native barcoding kit (1D EXP-NBD104) on an R9.4 SpotON flow cell with a MinION Mk1B device from Oxford Nanopore Technologies (ONT; UK). MinION was controlled with MinKNOW v3.1.19 (ONT). Base calling with the high-accuracy model and demultiplexing were accomplished by Guppy v3.1.5 (ONT) using default parameters. Porechop v0.2.3 (5) was used to trim adapters, applying default parameters, with additional internal adapter removal using a 90% identity threshold. Long-read sequencing of BL-3, BL-4, and BL-6 produced 465,840 reads (1.1 Gb of data, 270× coverage, *N*_50_ value of 9 kb), 186,991 reads (1.2 Gb, 500× coverage, *N*_50_ value of 11 kb), and 75,620 reads (0.5 Gb, 145× coverage, *N*_50_ value of 12 kb), respectively. Short-read sequencing of BL-3, BL-4, and BL-6 generated 2,272,799 reads (6.9 Gb, 180× coverage), 2,172,274 reads (6.6 Gb, 284× coverage), and 1,574,086 reads (4.8 Gb, 140× coverage), respectively.

Hybrid *de novo* genome assembly based on short and long reads was performed using Unicycler v0.4.8 with default parameters (6). The genome assembly of strain BL-3 resulted in seven contigs. For strains BL-4 and BL-6, single circular contigs were assembled. Putative gene coding sequences (CDSs) were identified and annotated using the MicroScope automatic annotation platform via external submission (7). The genome sizes, GC contents, numbers of predicted CDSs, and genome quality parameters are listed in Table 1. The new isolates and their genomes are valuable resources for exploring the metabolic features of chain-elongating bacteria.

**TABLE 1:** Genome features of isolates BL-3, BL-4, and BL-6

Feature	Data for strain:		
	BL-3	BL-4	BL-6
Genus assignment^*a*^	*Clostridium*_B	UBA4871	*Clostridium*_E
BioSample accession no.	SAMEA6567123	SAMEA6567124	SAMEA6567125
Run accession no.	ERR3959273, ERR3959415	ERR3959274, ERR3959416	ERR3959275, ERR3959417
WGS or chromosome accession no.	CADDXC010000000	LR778134	LR778135
Genome size (bp)	3,855,691	2,335,857	3,435,529
GC content (%)	34.32	42.75	54.63
No. of contigs	7	1	1
Completeness^*b*^ (%)	98.6	97.9	98.0
Contamination^*b*^ (%)	1.0	0.3	1.3
No. of CDSs	3,867	2,319	3,480
No. of tRNA genes	67 (21 types)	54 (20 types)	63 (21 types)
No. of rRNA genes (5S, 16S, 23S)	14 (4, 5, 5)	9 (3, 3, 3)	9 (3, 3, 3)
No. of miscellaneous RNA genes	524	24	32
No. of tmRNA^*c*^ genes	1	1	1

a Genus assignment refers to the Genome Taxonomy Database (8) phylogenomic classification.

b Genome completeness and contamination as calculated by CheckM (9).

c tmRNA, transfer-messenger RNA.

### Data availability.

The sequence data are available in the European Nucleotide Archive (ENA) database under accession number PRJEB36835; see Table 1 for the BioSample, WGS or chromosome, and run accession numbers.
